# Non-Kin Networks Matter More for Later-Born Cohorts of Unmarried Older Men’s Depressive Symptoms

**DOI:** 10.1093/geroni/igaf122.215

**Published:** 2025-12-31

**Authors:** Shannon Ang, Rahul Malhotra, Grace Chua

**Affiliations:** Nanyang Technological University, Singapore, Singapore; Duke-NUS Medical School, Singapore, Singapore; Nanyang Technological University, Singapore, Singapore

## Abstract

It is well-established that gender disparities in health are partially due to gender differences in social support network size and diversity – women are more likely to have better health outcomes because they tend to have larger and more diverse social support networks (i.e., including both kin and non-kin). Recent studies have shown cohort changes in network size and proportion of kin and non-kin members, but we know little about whether and how broad societal changes have altered the effect of social support networks on older adults’ well-being. Moreover, these societal changes are likely to differ between married and unmarried individuals’ well-being outcomes, since social support networks are altered through marriage. We explore cohort differences in how kin and non-kin social support networks are associated with depressive symptoms at the intersection of gender and marital status. We rely on six waves of data from two nationally representative surveys of older adults aged 60 years and above in Singapore. Results from growth curve models show that non-kin social support networks are more beneficial for unmarried men’s depressive symptoms, compared to married men. Additionally, this effect is stronger for later-born cohorts of unmarried men. Our findings highlight the need to revise our understanding of the effectiveness of kin and non-kin social support networks for later-born cohorts of older adults. Policy interventions that focus on older adults’ social support networks for better well-being will need to consider both non-kin and kin social support networks.

